# Changes in gut microbiota in the acute phase after spinal cord injury correlate with severity of the lesion

**DOI:** 10.1038/s41598-021-92027-z

**Published:** 2021-06-17

**Authors:** Gabriele Bazzocchi, Silvia Turroni, Maria Chiara Bulzamini, Federica D’Amico, Angelica Bava, Mirco Castiglioni, Valentina Cagnetta, Ernesto Losavio, Maurizio Cazzaniga, Laura Terenghi, Luisa De Palma, Giuseppina Frasca, Beatrice Aiachini, Sonia Cremascoli, Antonino Massone, Claudia Oggerino, Maria Pia Onesta, Lucia Rapisarda, Maria Cristina Pagliacci, Sauro Biscotto, Michele Scarazzato, Tiziana Giovannini, Mimosa Balloni, Marco Candela, Patrizia Brigidi, Carlotte Kiekens

**Affiliations:** 1 Neurogastroenterology Unit, Montecatone Rehabilitation Institute, via Montecatone 37, 40026 Imola, Bologna Italy; 2 Unit of Microbiome Science and Biotechnology, Department of Pharmacy and Biotechnology, University of Bologna, Bologna, Italy; 3 Spinal Unit, Montecatone Rehabilitation Institute, Imola, Bologna Italy; 4 Department of Medical and Surgical Sciences, University of Bologna, Bologna, Italy; 5 ASST Gaetano Pini CTO, Milan, Italy; 6 ICS Maugeri, Bari, Italy; 7 Ospedale Valduce, Costa Masnaga, Lecco, Italy; 8 Ospedale Policlinico, Bari, Italy; 9 ICS Maugeri, Pavia, Italy; 10 Ospedale Santa Corona, Pietra Ligure, Savona, Italy; 11 Ospedale Cannizzaro, Catania, Italy; 12 Azienda Ospedaliera, Perugia, Italy; 13 Fondazione Teresa Camplani, Domus Salutis, Brescia, Italy; 14 Montecatone Rehabilitation Institute, Imola, Bologna Italy

**Keywords:** Spinal cord diseases, Dysbiosis, Microbiome

## Abstract

After spinal cord injury (SCI), patients face many physical and psychological issues including intestinal dysfunction and comorbidities, strongly affecting quality of life. The gut microbiota has recently been suggested to influence the course of the disease in these patients. However, to date only two studies have profiled the gut microbiota in SCI patients, months after a traumatic injury. Here we characterized the gut microbiota in a large Italian SCI population, within a short time from a not only traumatic injury. Feces were collected within the first week at the rehabilitation center (no later than 60 days after SCI), and profiled by 16S rRNA gene-based next-generation sequencing. Microbial profiles were compared to those publicly available of healthy age- and gender-matched Italians, and correlated to patient metadata, including type of SCI, spinal unit location, nutrition and concomitant antibiotic therapies. The gut microbiota of SCI patients shows distinct dysbiotic signatures, i.e. increase in potentially pathogenic, pro-inflammatory and mucus-degrading bacteria, and depletion of short-chain fatty acid producers. While robust to most host variables, such dysbiosis varies by lesion level and completeness, with the most neurologically impaired patients showing an even more unbalanced microbial profile. The SCI-related gut microbiome dysbiosis is very likely secondary to injury and closely related to the degree of completeness and severity of the lesion, regardless of etiology and time interval. This microbial layout could variously contribute to increased gut permeability and inflammation, potentially predisposing patients to the onset of severe comorbidities.

## Introduction

A spinal cord injury (SCI) is a devastating event resulting in motor and/or sensory deficit, as well as vegetative dysfunction with huge implications for all aspects of the patient’s life and environment, and a high cost on society^1^. The clinical picture and functioning outcome depend on the level of injury and its completeness. Intestinal dysfunction is an almost inevitable occurrence after SCI of any etiology, traumatic and non-traumatic^2,3^. Alterations in motor and secretory functions and in digestion and absorption mechanisms can affect all segments of the digestive tract, from pharynx to the anal canal, but, incomprehensibly, only a few studies have investigated intestinal pathophysiology after SCI: only five papers have studied the alterations underlying clinically relevant events in the SCI patient, such as pathological gastroesophageal reflux resulting in airway inflammation and possibly pneumonia^4^.

Colorectal dysfunction is the most studied issue probably because defecation management greatly affects lifestyle and Quality of Life (QoL) in persons with SCI. On the one hand, the transport of contents through the colon is delayed and, on the other hand, continence and evacuation efficiency are impaired. Consequently, intestinal emptying is no longer complete and does not involve the same colonic tract as healthy subjects^5^. Persons with SCI and reduced mobility, having bowel motion may not be able to reach a toilet in time and episodes of fecal and/or gas incontinence or continuous soiling and leakage may occur. Persons with SCI report unsatisfactory bowel management as the primary cause of QoL impairment, to an even greater extent than problems regarding walking, bladder management and sexuality^6^. This awareness arises a while after injury, in relation to acceptance of new life conditions concerning mobility and autonomy^7,8^.

Studies investigating the colonic motor pattern after SCI are few and show contrasting results^9–11^. More recent studies concluded that intact neural transmission between the spinal cord and higher centers is not essential for normal colorectal responses to feeding and distension^12^. The peristaltic motor pattern producing massive ab-oral transport of contents consists of particularly intense contractions called High Amplitude Propagating Contractions (HAPC) that overlap the normal segmentation contractions and create the conditions, in the presence of feces and/or gas in the lumen, for evacuation to occur when socially convenient^13,14^. This propulsive contractile activity is absent in SCI patients^15^, particularly upon awakening, which distinguishes them from patients with Slow Transit Constipation of functional origin, in which HAPCs are less frequent, less vigorous, but still present^16,17^. The pathophysiological mechanisms that correlate lesion level and degree of completeness with the type of motor behavior of the different colonic segments are not fully understood, given their different innervation. However, it is established that the transport of solid and gaseous contents through the colon in people with SCI is pathologically slowed as a consequence of reduced motor and peristaltic activity, reduced visceral perception, anal sphincter dysfunction, inadequate abdominal muscle tone and the person’s reduced immobility. This causes incomplete evacuation, formation of fecal residues, fecal impaction and unwanted defecation and/or overflow fecal incontinence^18,19^.

The gastrointestinal tract, especially the colon, hosts a complex community of bacteria, but also viruses, fungi and parasites, i.e., the gut microbiota, which performs multiple crucial functions for homeostasis^20^. In addition to allowing the extraction of energy from otherwise indigestible carbohydrates and synthesizing vitamins, the gut microbiota modulates the development and functionality of the immune, endocrine and nervous systems, through the production of bioactive molecules that can enter the bloodstream and reach extra-intestinal organs^21^. Recent population studies showed that the Bristol Stool Form Scale (BSFS) score was among the strongest explanatory factors for gut microbiota variation^22,23^: stool consistency and frequency are associated with specific microbiota components and growth rates, suggesting that particular ecological networks are established depending on the context^24,25^. This is very relevant as other studies have shown that bolus size and consistency affect propulsion speed, thus indicating that propulsion in the large bowel is not a simple reflex but a more complex process involving an adaptable neuromechanical loop^26^. This may imply that an empty colon has no motor activity and that an altered microbiota composition, i.e., dysbiosis, has a profound role in the pathophysiology of colon disorders, as already shown in several functional syndromes, such as chronic constipation, Irritable Bowel Syndrome and other colon motility disorders^27–29^. Investigating variations in the composition of the intestinal microbiota after SCI, in consideration of its deep relationship with Enteric Nervous System functionality and, consequently, colonic motor activity^30,31^ constitutes an important element to understand the pathophysiology of intestinal dysfunctions in SCI patients. Interestingly, following an experimental injury in mice, it was shown that the perilesional area is significantly larger in the presence of severe dysbiosis, which exacerbates neurological impairment and spinal cord pathology after SCI^32^. Not least, peculiar dysbiotic profiles may favor the onset of sepsis and organ infectious complications, so frequent in the clinical course after SCI. Dysbiosis in critically ill patients, such as after severe and sudden insults^33^, can lead to sepsis, from Systemic Inflammatory Response Syndrome to the almost always fatal Multi Organ Dysfunction Syndrome^34–36^. According to Wallace and colleagues, trauma in its various forms is likely to cause dysbiotic changes in the gut microbiota, which are strongly influenced by the variety of local and systemic phenomena, and in turn act pathologically, favoring the onset and severity of comorbidities. With specific regard to SCI, the authors point out that understanding how the gut microbiome changes following injury and whether it has a role in potentiating inflammation or protecting against secondary injury and infections, holds great promise for the development of microbiota-targeted intervention strategies to enhance patient care and potentially improve outcomes^37^.

To our knowledge, only Gungor et al.^38^ and Zhang et al.^39^ investigated the composition of the gut microbiota in SCI patients. The former assessed the microbiota in 30 patients ≥ 12 months after traumatic SCI (15 with complete lesion, above T6 level, and 15 with cauda equina syndrome), compared to 10 healthy subjects, all males of the same age. The latter evaluated 43 male patients at least 6 months after complete traumatic SCI (20 tetraplegics and 23 paraplegics), compared to 23 healthy male adults, aged 18–60 years. Both studies documented the presence of dysbiosis after SCI and suggested that it may play a role in neurological recovery.

The primary objective of the present study was to characterize the composition of the intestinal microbiota in a larger population of patients, of both genders, with traumatic and non-traumatic SCI, in the acute and early post-acute phase (< 60 days from SCI), as compared with a healthy Italian population of overlapping age, gender and geographical origin. Secondary objectives were to correlate the microbiota composition with patient characteristics (gender, age, intestine characteristics, Body Mass Index—BMI), type of SCI (traumatic or non-traumatic etiology, lesion level and completeness), spinal unit location, nutrition and concomitant antibiotic therapies.

## Results

### Study cohort description

One hundred SCI patients were enrolled from Spinal Units and Neuro-Rehabilitation Centers in nine different Italian cities: Bari, Brescia, Catania, Costa Masnaga, Milan, Imola, Pietra Ligure, Pavia and Perugia (Supplementary Fig. S1). The number of patients enrolled is evenly distributed among the North (31), Center (30) and South (39) of the country, so that dissimilarities due to different regional eating habits/lifestyles can possibly be identified.

Demographic, neurological and clinical characteristics of enrolled SCI patients are shown in Table 1. As expected, the majority of patients are male and etiology is traumatic. The mean age is very similar. The percentages of patients in the three groups according to lesion level and in the four related to AIS score, allow a meaningful comparison among them (please, see also “Methods”). In accordance with inclusion criteria, the average time interval between injury and fecal sampling is about one month with minimal variation among centers.

**Table 1:** Demographic, neurological, anthropometric and clinical characteristics of the 100 enrolled Italian patients with SCI.

Spinal unit												
		Imola	Pavia	Brescia	Pietra L	Bari^a^	Catania	Perugia	Milan	Bari^b^	Costa M	
Pts (no.)		10	8	8	10	12	16	10	7	11	8	100
Male (%)		100	75	87.5	60	66.6	68.7	100	57.1	63.6	75	76%
Age, years (mean ± SD)		45 ± 14	51 ± 20	51 ± 14	53 ± 13	50 ± 19	54 ± 13	47 ± 15	60 ± 12	49 ± 14	43 ± 21	51 ± 15
Traumatic etiology (%)		100	87.5	87.5	70	75	68.7	30	28.5	63.6	62.5	68%
Neurologic lesion level	C1–C6 (%)	20	25	12.5	30	58.3	31.2	40	28.5	18.8	12.5	29%
	C7–T5 (%)	40	25	25	60	0	6.3	20	43	18.8	37.5	25%
	T6–L5 (%)	40	50	62.5	10	41.7	62.5	40	28.5	63.4	50	46%
AIS score	A (%)	70	50	37.5	30	50	12.5	10	43	18.2	62.5	36%
	B (%)	0	25	25	20	0	12.5	30	14.3	27.2	12.5	16%
	C (%)	0	25	37.5	50	41.6	43.7	10	14.3	54.6	12.5	31%
	D (%)	30	0	0	0	8.4	31.3	50	28.4	0	12.5	17%
Time interval between lesion and fecal sampling	Days ± SD	21 ± 8	30 ± 33	18 ± 10	34 ± 16	32 ± 16	29 ± 17	23 ± 18	36 ± 22	19 ± 4	28 ± 1	27 ± 5
	1–15 (%)	20	50	50	20	27.3	25	40	14.3	18.8	25	27%
	16–30 (%)	70	12.5	37.5	10	27.3	43.7	40	28.7	81.2	50	41%
	31–60 (%)	10	37.5	12.5	70	54.4	31.3	10	57	0	25	31%
BMI	< 18.5 (%)	0	12.5	0	0	0	6.3	0	14.3	0	12.5	4%
	≥ 18.5 ≤ 24.9 (%)	50	62.5	62.5	100	66.7	37.5	70	71.4	91	62.5	66%
	≥ 25 (%)	50	25	37.5	0	33.3	56.2	30	14.3	9	25	30%
Nutrition	OS (%)	0	75	50	50	75	93.7	70	100	81.8	62.5	67%
	EN and/or PN (%)	100	25	50	50	25	6.3	30	0	18.2	37.5	33%
BSFS	Type 1–3 (%)	0	0	50	40	33.4	50	0	42.8	36.6	37.5	30%
	Type 4–5 (%)	80	37.5	50	60	66.6	50	100	57.2	54.4	37.5	60%
	Type 6–7 (%)	20	62.5	0	0	0	0	0	0	9	25	10%
Antibiotic intake	No (%)	70	62.5	50	40	33.4	18.7	30	28.5	36.3	50	40%
	Yes, 3 days (%)	10	25	50	50	66.6	50	40	43	36.3	37.5	42%
	Yes, 10 days (%)	20	12.5	0	10	0	31.3	30	28.5	27.4	12.5	18%

As for the time interval between lesion and fecal sampling, patients were grouped into the following categories: ≤ 15 days, between 16 and 30 days, and between 31 and 60 days. As for BMI, patients were divided into the following categories: underweight (BMI < 18.5 kg/m^2^), normal weight (BMI between 18.5 and 24.9 kg/m^2^) and overweight/obesity (BMI ≥ 25 kg/m^2^). *OS* per os nutrition, *EN* enteral nutrition, *PN* parenteral nutrition, *BSFS* Bristol Stool Form Scale. With regards to the antibiotic intake, “No” stands for untreated patients in the past 10 days, “Yes, 3 days” for patients who were given antibiotics in the 3 days before fecal sampling, “Yes, 10 days” for patients who were given antibiotics between 4 and 10 days before fecal sampling. In the last column the total of patients and the average in years/days and in percentages per each variable/group are reported.

^a^Ospedale Policlinico.

^b^ICS Maugeri.

### The gut microbiota of SCI patients segregates from that of healthy controls

The gut microbiota of SCI patients was profiled by 16S rRNA gene-based next-generation sequencing and compared with that of age- and sex-matched healthy Italians (aged 45.8 ± 14.1 years), whose data are publicly available^40–43^. A total of 1,171,676 high-quality reads (mean ± SD, 11,717 ± 6088) were generated and clustered into 12,442 OTUs at 97% identity.

**Figure 1:**
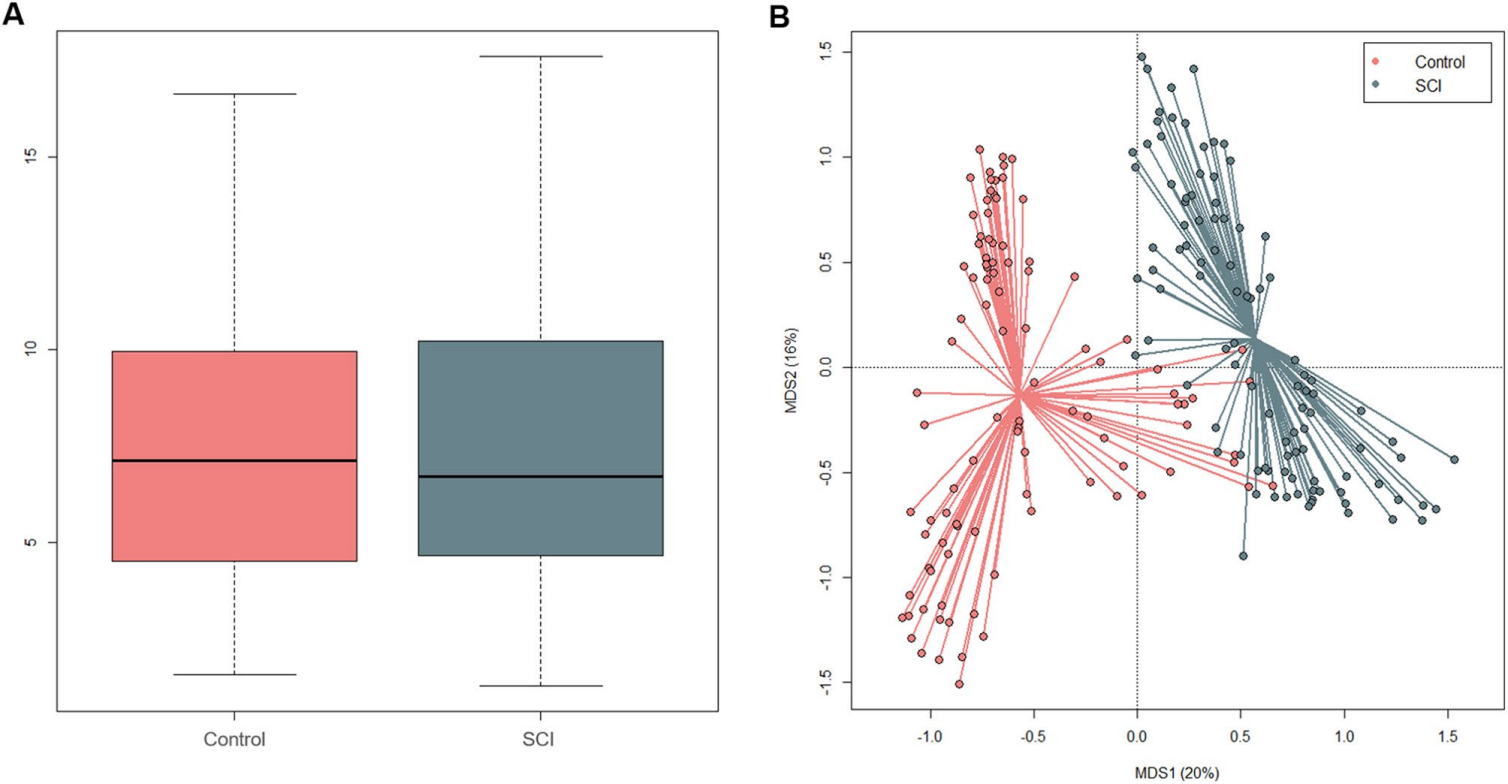
Diversity of the gut microbiota of SCI patients as compared to healthy controls. (**A**) Box plots showing the distribution of alpha diversity values, according to the inverse Simpson index in SCI patients as compared to age- and sex-matched healthy Italian controls. (**B**) Principal Coordinates Analysis based on Bray–Curtis distances between the genus-level microbial profiles of SCI patients and healthy controls. A significant separation was found (p = 0.001, permutation test with pseudo-F ratios).

No differences were observed in alpha diversity between SCI patients and healthy controls (p = 0.8, Wilcoxon rank sum test) (Fig. 1A). On the other hand, the Principal Coordinates Analysis (PCoA) of inter-individual variation, based on Bray–Curtis distances between the genus-level profiles, revealed a significant separation between the study groups (p = 0.001, R^2^ = 0.15; permutation test with pseudo-F ratios) (Fig. 1B). This data was confirmed by Random Forests (error rate at discriminating SCI patients vs. healthy controls, 1%).

**Figure 2:**
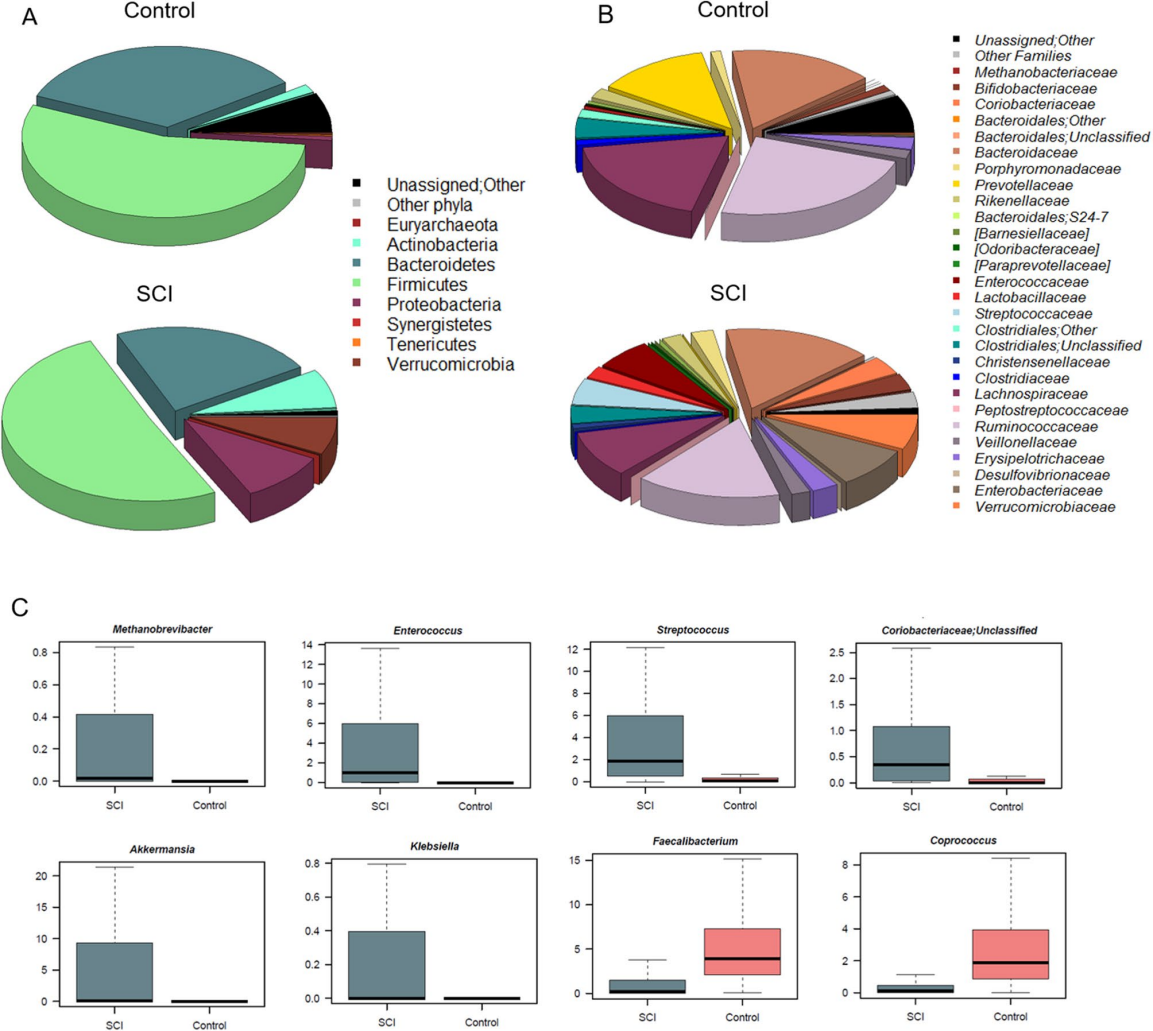
Compositional structure of the gut microbiota in SCI. Pie charts showing the average relative abundance of the most abundant phyla (**A**) and families (**B**) in SCI patients as compared to age- and sex-matched healthy Italian controls. Only taxa with relative abundance > 0.1% in at least 20 samples for phyla and > 0.01% in at least 30 samples for families are shown. (**C**) Box plots showing the distribution of the relative abundance values of discriminant genera between SCI patients and healthy controls, according to Random Forests. p < 0.001, Wilcoxon rank sum test.

**Figure 3:**
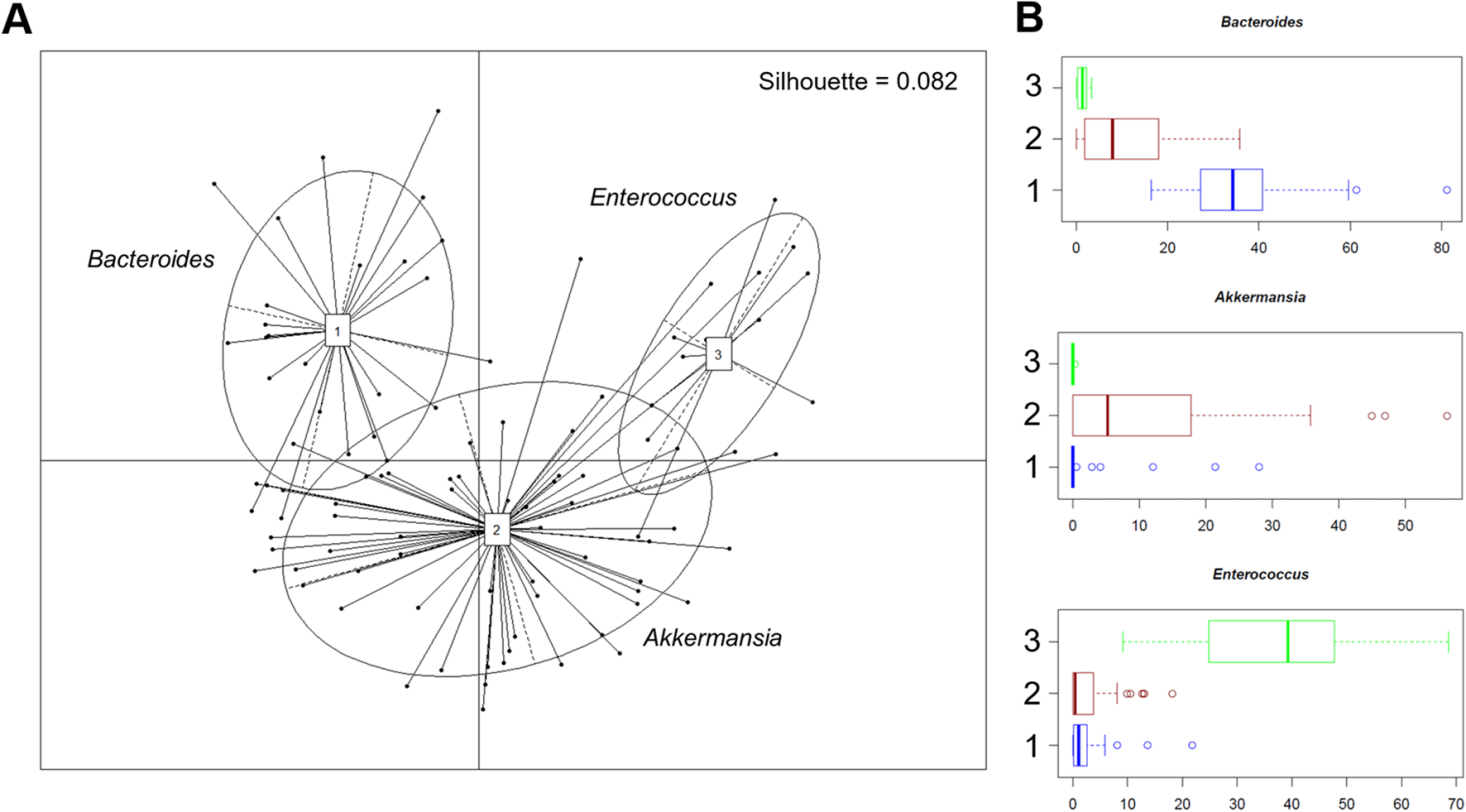
Enterotype-like groups in SCI patients. (**A**) Principal Coordinates Analysis of enterotype clusters in SCI patients. Three enterotypes were identified by using the Jensen-Shannon divergence distance metric. The ellipse covers 67% of the samples belonging to a cluster. The silhouette coefficient is shown at the top right. (**B**) Box plots showing the distribution of the relative abundance values of discriminant genera among the three enterotype-like groups. p < 0.001, Kruskal–Wallis test.

In line with the available literature^44^, the microbial profiles of both SCI patients and healthy controls are dominated by Firmicutes (mean relative abundance, SCI vs. controls: 50.9% vs. 54.8%) and Bacteroidetes (22.8% vs. 34.3%), with the families *Ruminococcaceae* (15.5% vs. 23.6%), *Lachnospiraceae* (9.4% vs. 18.5%) and *Bacteroidaceae* (16.1% vs. 16.1%) being the most represented regardless of health status (Fig. 2A,B). Nevertheless, many differences emerged, suggesting an altered composition of the gut bacterial community after SCI. In particular, SCI patients are enriched in several families variously involved in inflammation-based disorders^45–48^, such as *Coriobacteriaceae* (3.8% vs. 0.5%), *Enterococcaceae* (6.3% vs. 0.03%), *Lactobacillaceae* (2.5% vs. 0.2%), *Streptococcaceae* (5.4% vs. 0.6%), *Methanobacteriaceae* (0.3% vs. 0.002%) and *Enterobacteriaceae* (8.3% vs. 0.5%), as well as *Verrucomicrobiaceae* (7.2% vs 0.4%) (p < 0.001, Wilcoxon rank sum test). Moreover, they showed a decrease in *Prevotellaceae* (0.7% vs. 12.6%), *Clostridiaceae* (0.6% vs 1.0%) and the short-chain fatty acid (SCFA)-producing family, *Ruminococcaceae* (15.5% vs. 23.6%) (p < 0.001) (Fig. 2B). Consistent data were obtained at the genus level and confirmed by Random Forests, resulting in the identification of the following highly discriminatory taxa: *Methanobrevibacter* (0.3% vs. 0.002), an unclassified genus belonging to *Coriobacteriaceae* (0.8% vs. 0.1%), *Streptococcus* (5.2% vs. 0.6%), *Enterococcus* (6.3% vs. 0.03%), *Klebsiella* (3.0% vs. 0.05%), *Akkermansia* (7.2% vs. 0.4%), *Faecalibacterium* (1.1% vs. 5.2%), and *Coprococcus* (0.5% vs. 2.8%) (p < 0.001) (Fig. 2C). Interestingly, enterotype analysis revealed that SCI patients could be stratified into three distinct groups, based on the Jensen-Shannon distance metrics, which were dominated by *Bacteroides* (enterotype-like group 1), *Akkermansia* (enterotype-like group 2) and *Enterococcus* (enterotype-like group 3), respectively (p < 0.001, Kruskal–Wallis test) (Fig. 3). The vast majority (64%) of samples fell into group 2, 26% into group 1 and the remaining 10% into group 3.

### The gut microbiota profiles of SCI patients stratify by lesion level and AIS score

**Figure 4:**
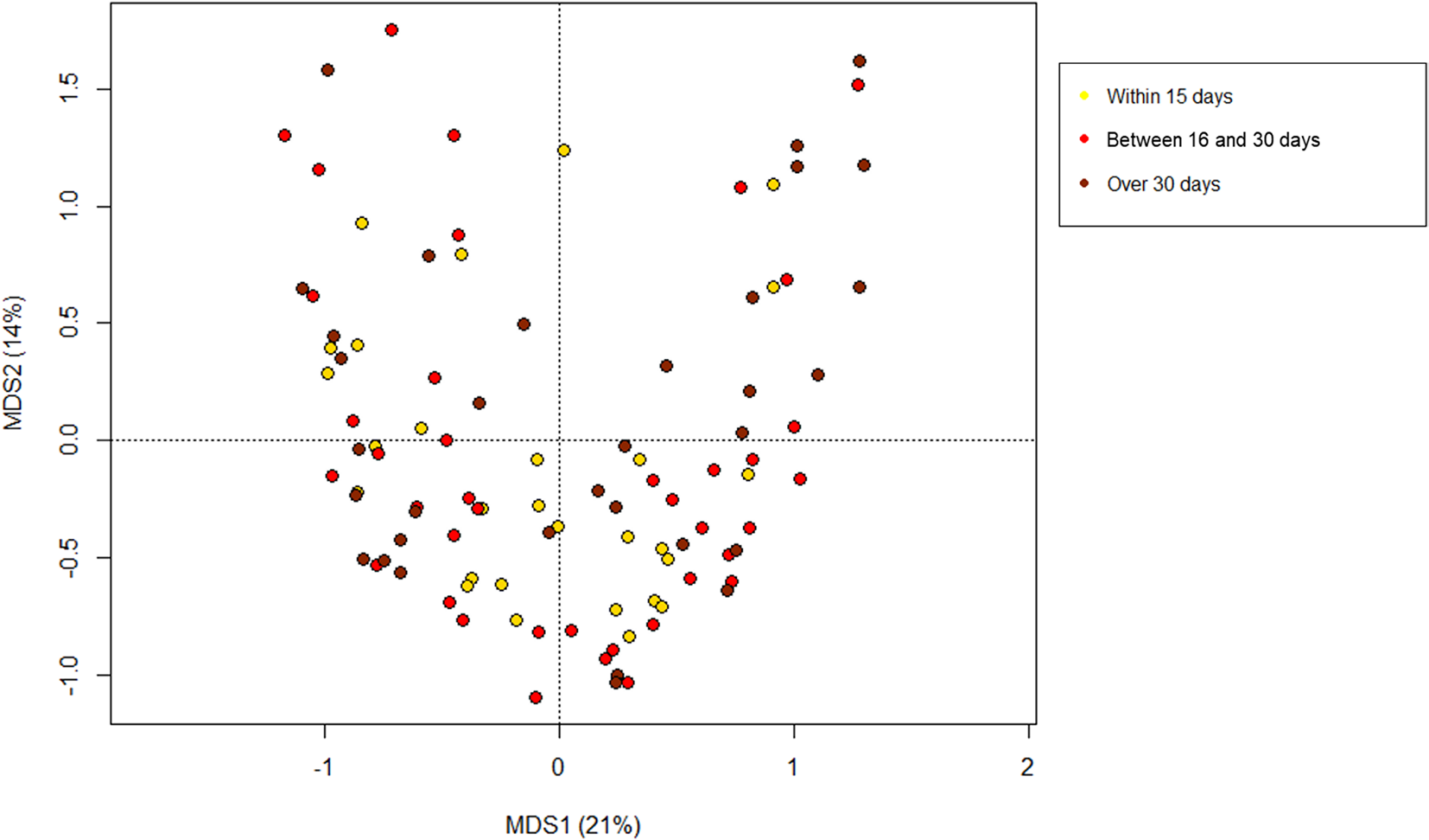
The gut microbiota dysbiosis in SCI patients is stable over time. Principal Coordinates Analysis based on Bray–Curtis distances between the genus-level microbial profiles of SCI patients according to the time interval between injury and fecal sampling (≤ 15 days, between 16 and 30 days, over 30 days). No significant segregation was found (p > 0.05, permutation test with pseudo-F ratios).

PCoA analysis based on Bray–Curtis distances between the genus-level profiles of SCI patients showed no segregation by time interval since the injury (≤ 15 days, between 16 and 30 days, over 30 days) (p > 0.05, permutation test with pseudo-F ratios) (Fig. 4), suggesting that microbiota dysbiosis is unrelated to the tremendous metabolic and neurological conditions that characterize spinal shock, which usually ends within the first 20–30 days after the lesion. Similarly, no separation was observed by well-known variables to be associated with the gut microbiota^22,43,49,50^, i.e. age, BMI (under vs. normal vs. overweight/obesity), type of nutrition (per os vs. enteral/parenteral) and BSFS (1–3, 4–5, 6–7), nor gender or etiology of the lesion (traumatic vs. non-traumatic) (p > 0.05) (Supplementary Figs. S2–S7). However, a few microbial signatures consistent with literature were highlighted, including lower representation of Bacteroidetes members and *Faecalibacterium*, and enrichment of enterobacteria in patients following enteral/parenteral nutrition vs. those fed per os (p ≤ 0.04, Wilcoxon rank sum test) (Supplementary Fig. S4)^51^. As for BMI, underweight SCI patients were characterized by increased proportions of *Dialister* and *Fusobacterium*, overweight ones by increased amounts of *Enterococcus*, while *Prevotella* was distinctive of normal-weight patients (p ≤ 0.03) (Supplementary Fig. S3).

A slight separation in the ordination space was observed by recruitment center (p = 0.003, R^2^ = 0.13; permutation test with pseudo-F ratios) (Supplementary Fig. S8). In particular, the samples from the spinal unit of Pietra Ligure form a cluster slightly separated from the others (p ≤ 0.04, R^2^ = 0.03). A barely separable grouping was also observed for Catania, with respect to Bari (Ospedale Policlinico), Costa Masnaga, Milan and Imola (p ≤ 0.04, R^2^ ≤ 0.02). Random Forests-based analysis confirmed the data, i.e., that the rehabilitation center does not strongly affect the patients’ microbiota (error rate at discriminating between centers, 83%), with only the samples from Catania being quite accurately discriminated (i.e., 10/16). Such a discrimination is mainly related to a lower representation of *Streptococcus* and *Faecalibacterium* while greater proportions of *Bacteroides* and *Rikenellaceae* in the Catania-derived samples compared to the other spinal units (p ≤ 0.04, Kruskal–Wallis test) (Supplementary Fig. S8). According to the enterotype analysis, 7/16 patients from Catania fell in fact in the enterotype-like group 1 dominated by *Bacteroides*.

**Figure 5:**
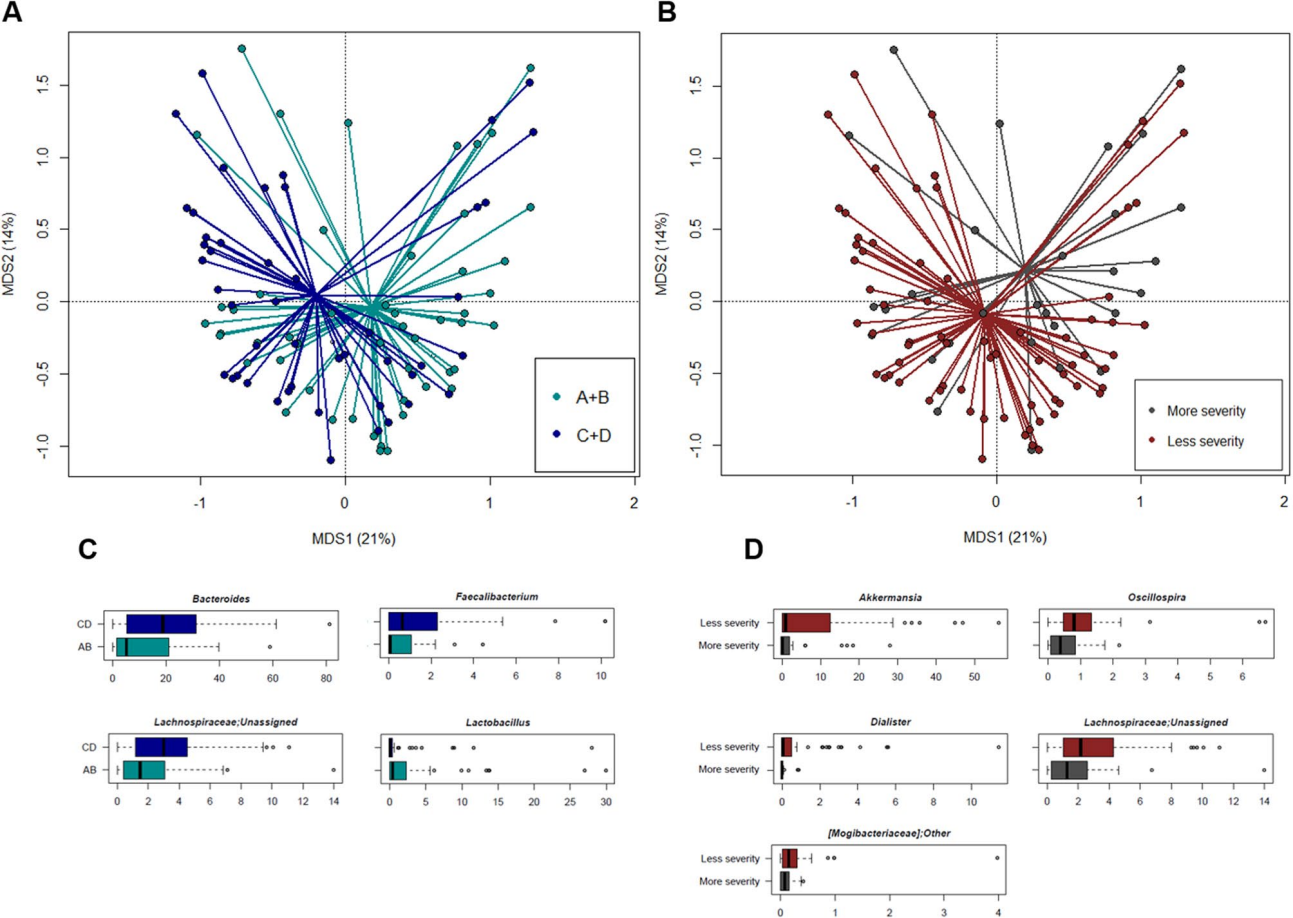
The gut microbiota profiles of SCI patients stratify by AIS score and severity of injury. Principal Coordinates Analysis based on Bray–Curtis distances between the genus-level microbial profiles of SCI patients according to AIS score (**A**) and SCI severity (**B**). A significant separation by both covariates was found (p ≤ 0.04, permutation test with pseudo-F ratios). More severity, AIS score A or B and neurological level of lesion between C1 to C6 and C7 to T6; less severity, AIS score A or B and lesion level between T7 and L5, and AIS score C or D and every neurological level of lesion. Box plots showing the distribution of the relative abundance values of discriminatory genera by AIS score (**C**) and SCI severity (**D**). p ≤ 0.05, Wilcoxon rank sum test.

Interestingly, the genus-level layouts of SCI patients were found to stratify by AIS score, i.e., there was a significant separation between patients with AIS A and B vs. those with AIS C and D (p = 0.03, R^2^ = 0.03; permutation test with pseudo-F ratios) (Fig. 5A). Significant segregation was also found by injury severity: more severity for patients with AIS A or B and neurological level of lesion between C1 to C6 and C7 to T6, vs. less severity for patients with AIS A or B, but lesion level between T7 and L5 together with patients with AIS C or D and any neurological level of lesion (p = 0.04, R^2^ = 0.02) (Fig. 5B). At the taxonomic level, the genera *Bacteroides* and *Faecalibacterium*, and unclassified members of *Lachnospiraceae* were overrepresented in patients with grade C or D impairment, whereas *Lactobacillus* was more abundant in AIS grade A or B patients, especially in those with a C1–C6 cervical lesion (p ≤ 0.04, Wilcoxon rank sum test) (Fig. 5C). In the latter patient subset, *Lactobacillus* OTUs were variously assigned to the species *L. rhamnosus*, *L. gasseri*, *L. helveticus*, *L. fermentum* and *L. mucosae*. As for injury severity, the proportions of the mucus degrader *Akkermansia*, as well as those of *Oscillospira*, *Dialister*, and unclassified genera of *Lachnospiraceae* and [*Mogibacteriaceae*] were reduced in the group having more severe SCI (p ≤ 0.05) (Fig. 5D). When looking at the enterotype distribution, we found that although the enterotype-like group 2 (dominated by *Akkermansia*) was the most represented in both patients with more (59%) and less injury severity (66%), the enterotype-like group 3 (dominated by *Enterococcus*) accounted for a greater percentage of those having more severe SCI (18% vs 7% in patients with less severe injury). It should be noted that more than half of all patients had taken antibiotics but this percentage was higher in those with more severe SCI (76% vs 56% in those with less severe injury).

**Figure 6:**
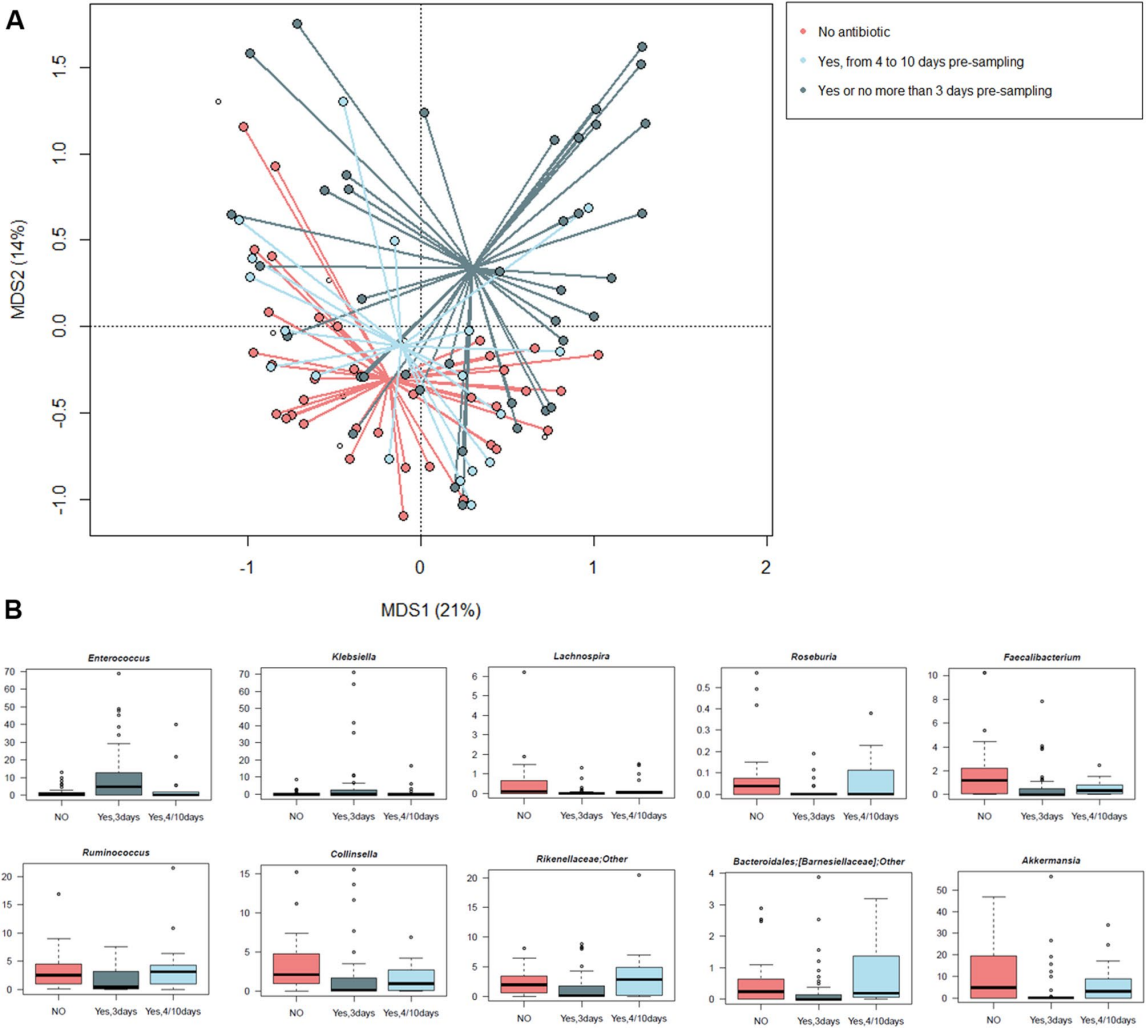
Impact of antibiotics administration on the gut microbiota of SCI patients. (**A**) Principal Coordinates Analysis based on Bray–Curtis distances between the genus-level microbial profiles of SCI patients according to antibiotics administration and timing (i.e., no antibiotic therapy, and therapy in the 3 days or between 4 and 10 days before fecal sampling). A significant separation among groups was found (p = 0.001, permutation test with pseudo-F ratios). (**B**) Box plots showing the distribution of the relative abundance values of discriminant genera. p ≤ 0.05, Kruskal–Wallis test.

As expected, a significant segregation was found between the gut microbiota structures of SCI patients who had or not been given antibiotic therapy, with a further stratification by timing of administration (i.e., in the 3 days or between 4 and 10 days before fecal sampling) (p = 0.001, R^2^ = 0.07; permutation test with pseudo-F ratios) (Fig. 6A). In particular, the proportions of *Enterococcus* and *Klebsiella* were far greater in patients who had received antibiotics in the 3 days before sampling (p ≤ 0.03, Kruskal–Wallis test). *Enterococcus* and *Klebsiella* OTUs were mainly assigned to *E. faecium* and *K. pneumoniae*, respectively. On the other hand, typically health-associated microbiota components, such as *Lachnospiraceae* and *Ruminococcaceae* members, were greatly reduced by recent antibiotics administration, but their levels after a few days (i.e., 4–10) were overall comparable to those of patients who had not taken therapy (p ≤ 0.02). A similar trend was observed for *Collinsella*, Bacteroidetes members (i.e., *Rikenellaceae* and [*Barnesiellaceae*]) and *Akkermansia* (p ≤ 0.01) (Fig. 6B). As expected, the *Enterococcus* enterotype (group 3) was particularly represented in patients who had received antibiotics in the 3 days before sampling (24%) but its proportion dropped after 4–10 days (6%), up to 0% in those who had not received antibiotics.

## Discussion

To the best of our knowledge, this is the first study on the intestinal microbiota in acute and early post-acute SCI patients, some of them still in a phase of spinal shock, being in the first 10–12 weeks after injury. Consistently with Gungor et al.^38^ and Zhang et al.^39^, their gut microbiota appears dysbiotic and shows distinct signatures compared to age- and sex-matched healthy controls from the same geographical location. In addition to a reduction in beneficial SCFA producers (e.g. *Faecalibacterium* and *Coprococcus*), which probably represents a non-specific, shared response to diseases^52^, SCI patients show increased proportions of potentially pathogenic and pro-inflammatory bacteria, such as *Enterococcus*, *Streptococcus* and *Klebsiella*. These include well-known nosocomial pathogens, associated with urinary tract infections, bacteremia, and elevated rates of mortality in critically ill patients^36,53,54^. As expected, *Enterococcus* and *Klebsiella* were more abundant in patients who had received antibiotic therapy in the 3 days prior to fecal sampling. According to previous findings, their bloom could be closely related to the drop in oxidative stress-sensitive SCFA-producing commensals, with increased epithelial oxygenation and thus oxygen availability for facultative anaerobic pathogens^55,56^. Moreover, SCI patients were enriched in *Methanobacteriaceae*, *Coriobacteriaceae* and *Verrucomicrobiaceae*, specifically *Akkermansia*. The archaeal species *Methanobrevibacter smithii* was found to increase in critically ill patients, possibly due to its intrinsic resistance to antibiotics^54^. However, it should be noted that the primer combination we used was shown to be not optimal for Archaea detection^57^, which may have biased our results. On the other hand, *Coriobacteriaceae* members have been shown to reduce the expression of tight-junction proteins, possibly leading to gut leakage and metabolic endotoxemia^58^. The role of the mucus degrader *Akkermansia* is instead more controversial, since it has been proposed as next-generation probiotics for metabolic disorders^59^, but also shown to induce pro-inflammatory T lymphocyte responses and potentially exacerbate the symptoms of neurodegenerative disorders, such as Multiple Sclerosis and Parkinson’s disease^60,61^. It is plausible that in a compromised clinical state, high amounts of *Akkermansia* contribute to the erosion of the mucus layer, compromising the barrier function and facilitating access to epithelia by enteropathogens, with consequently increased inflammatory tone.

Apart from the decrease in SCFA producers, the discriminatory taxa herein identified do not overlap with the results by Gungor et al.^38^ and Zhang et al.^39^. The reasons may include technical issues related to library preparation and bioinformatics, exiguity of their patient population, ethnicity and geographic location (including lifestyle, environmental exposure, socio-economic status, etc.)^23,62^ and, not least, the chronicity of SCI in their population. Furthermore, patients who had taken antibiotics and/or probiotics in the previous 3 weeks^38^ and month^39^ were excluded, so it was not possible to make between-study comparisons on the impact of the antibiotic intake on the intestinal microbiota of SCI patients.

The gut microbiota dysbiosis in SCI is robust to several variables known to be associated with the microbiota variation, i.e., age, gender, BMI, type of nutrition and BSFS^23,43,49,50,63^, thus corroborating the hypothesis that the onset of dysbiosis is secondary to SCI. On the other hand, our data showed an impact, albeit limited, of the spinal unit, with the Catania-derived samples being more easily discriminated against by others. It is not unusual to find inter-hospital differences, possibly related to the hospital environment itself, not only food related, as previously discussed^64^. While caution must be taken in associating discriminatory taxa with other variables, in the light of the data commented above we believe that the center-related variation should not have biased our results.

No differences were found in relation to time since injury as well as etiology, be it traumatic or non-traumatic. The previous studies and obviously also the animal studies with experimental lesions, concerned only traumatic injuries so this is the first demonstration that dysbiosis is associated with SCI of any origin. In contrast, we found that the gut microbiota is significantly different between AIS A or B patients, i.e., with motor complete lesion, and patients with motor incomplete injury, i.e., AIS C or D. The previous studies^38,39^ had not explored this correlation, because only complete patients were included, however the need to verify these data had already been reported^65^. In particular, the proportions of *Bacteroides*, *Faecalibacterium* and *Lachnospiraceae* are reduced in complete patients, suggesting further loss of health-promoting microorganisms and reduced bioavailability of SCFAs. On the contrary, in these patients *Lactobacillus* is more present. *Lactobacillus* spp. are low-virulence subdominant commensals, widely used as probiotics; however, this genus and/or certain species have also been found to be overabundant in some inflammatory disorders, including obesity, coronary heart disease and heart failure^66–68^. Lactobacilli are also frequently resistant to vancomycin, which could explain their persistence under critical health conditions^69^.

The division of patients into a group with severe SCI, i.e., AIS A or B and C1-T6 lesion level, and a less neurologically compromised group, i.e., AIS A or B with T6-L5 lesion level and all AIS C or D, confirmed that the severity of intestinal dysbiosis is related to the severity of the neurological damage. In particular, the under-representation of health-associated taxa, such as *Lachnospiraceae*, *Mogibacteriaceae* and *Oscillospira*^52,70,71^, in more severe SCI further stresses the presence of an unbalanced bacterial ecosystem with reduced SCFA production capacity, potentially contributing to increased intestinal permeability and inflammation. *Oscillospira* is able to utilize host glycans as well as *Akkermansia*, whose relative abundance was also found to be reduced in more severe vs. less severe SCI. In addition, the most neurologically impaired patients showed reduced proportions of *Dialister*, a lactate-utilizing genus inversely related to inflammation and recently proposed as a potential lead for psychobiotics^72^. Interestingly, *Dialister* was also found to be lower in complete SCI in both Turkish^38^ and Chinese patients^39^. On the other hand, the enterotype analysis showed that the enterotype-like group 3 (*Enterococcus* dominated) was far more represented in patients with more severe SCI, suggesting a possible involvement of this pathobiont. As discussed above, this could be closely related to antibiotic intake, as a high percentage of patients with more severe injury had taken antibiotics, particularly in the 3 days prior to fecal sampling. The reasons for the variations in these taxa remain unclear and will require further investigation. Given the recent acquisition of knowledge about the important role of the intestinal microbiome in neuropathic and visceral pain^73,74^, it would have been interesting to stratify the microbiota data also in relation to pain symptoms, but the sample size for every different neurologic damage was too small to achieve meaningful results. Moreover, this study regards the very early post-acute phase after SCI and for the study of neuropathic pain it would be too early, as neuropathic pain usually develops up to 6 months after onset.

In summary, patients in the acute phase after SCI are characterized by distinctive alterations of the intestinal microbiota, which appear to be stable at least during the first 60 days of injury. Dysbiosis does not vary by gender, age, BMI, type of hospital nutrition, location of the spinal unit where hospitalized, BSFS or etiology. In contrast, it varies according to the degree of completeness and severity of the lesion: patients with tetraplegia and motor complete lesions show a more compromised gut microbiota profile. However, these data may be partially biased by antibiotic intake and need to be validated in larger independent cohorts.

Once confirmed, this knowledge may help to better understand and hopefully predict the onset and severity of common post-injury comorbidities, such as sepsis, organ infection, immunological break-down, catabolic condition, intestinal and bladder complications, by clarifying the reasons why the recovery of some patients is more impaired than others.

## Methods

### Patients and fecal sampling

We scheduled to enroll around 100 consecutive patients from 10 Italian Spinal Units and Centers for Neurologic Rehabilitation, non-competitively, according to the following inclusion criteria:

- age ≥ 18 and ≤ 70 years, both male and female
- traumatic or non-traumatic SCI following vascular myelopathy or transverse myelitis
- onset within 60 days
- SCI of any neurological level ≥ L5, complete or incomplete, classifiable as grade AIS A, B, C, D. The International standards for neurological classification of SCI represent the gold standard assessment for documentation of the level and severity of SCI^75^. The lesion completeness is defined according to the American Spinal Injury Association Impairment Scale (AIS) with the following grades: AIS A: complete, AIS B: sensory incomplete, AIS C and D: motor incomplete and AIS E: normal function^76^.
- no more than 7 days since first admission to Spinal Unit.

Exclusion criteria were: non-traumatic etiology different from vascular myelopathy or transverse myelitis (others causes for SCI (neoplasms, discitis, etc.) do not guarantee acute onset and precise lesion level), presence of pre-existing intestinal pathologies such as Inflammatory Bowel Disease, chronic hepatitis, Celiac Disease, neoplasm, extensive intestinal resection, ongoing diarrhea (more than 6 watery evacuations per day and/or fecal volume > 250 ml over 24 h), intake of probiotics after injury, ongoing severe sepsis, pregnancy, brain injury and/or coma status associated with SCI, and inability to give informed consent.

A single fecal sample was collected for each patient during a spontaneous or scheduled evacuation, within the first week of hospitalization at the rehabilitation center. Samples were stored at − 20 °C and sent under temperature-controlled conditions from all Centers to the same Microbiology Laboratory, where they were kept at − 80 °C until analysis.

### Endpoints

For the Primary Objective: deviation of the gut microbiota profile of patients with SCI from that of the healthy population living in the same geographical area, matched by gender and age.

For the Secondary Objective: correlation between the gut microbiota profile of SCI patients and sex, age, traumatic and non-traumatic etiology, lesion level, AIS score, interval between injury and fecal sampling, BMI at admission, type of nutrition, stool type at the time of sampling and any previous or ongoing antibiotic therapy. As for lesion level, patients were arbitrarily divided into 3 groups according to the severity of sensory-motor impairment and integrity of the Sympathetic Nervous System: C1–C6 group includes high tetraplegia; C7–T6 includes tetraplegia with residual upper limb function and paraplegia with impairment of the sympathetic pathways and involvement of abdominal muscles; T7–L5 injuries result in paraplegia without sympathetic involvement of a part for the large bowel. Regarding the time interval from the injury, in order to evaluate the potential impact of the spinal shock, which usually ends within the first 20–30 days after the lesion, we selected the following cut-off values: until 15 days, between 16 and 30 days, and over 30 days.

### Microbial DNA extraction and 16S rRNA gene sequencing

Microbial DNA was extracted from approximately 250 mg of fecal sample using the repeated bead-beating plus column method as previously reported^77^. Briefly, the samples were suspended in 1 mL of lysis buffer (500 mM NaCl, 50 mM Tris–HCl pH 8, 50 mM EDTA, 4% SDS) and processed three times in a FastPrep instrument (MP Biomedicals, Irvine, CA) at 5.5 movements/s for 1 min, in the presence of four 3-mm glass beads and 0.5 g of 0.1-mm zirconia beads (BioSpec Products, Bartlesville, OK). Stool particles were pelleted after 15 min of incubation at 95 °C, and nucleic acids were precipitated by adding 10 M ammonium acetate and one volume of isopropanol. The pellets were then washed with 70% ethanol and resuspended in 10 mM Tris–HCl, 1 mM EDTA pH 8.0 (TE) buffer. After treatment with 10 mg/mL DNase-free RNase at 37 °C for 15 min, the samples were subjected to protein removal and column-based DNA purification following the manufacturer’s instructions (DNeasy Blood & Tissue kit; QIAGEN, Hilden, Germany).

The V3–V4 hypervariable region of 16S rDNA was amplified by using the 341F and 785R primers with added Illumina adapter overhang sequences as previously described^48^. After amplicon purification with a magnetic bead-based clean-up system (Agencourt AMPure XP; Beckman Coulter, Brea, CA), indexed libraries were prepared by limited-cycle PCR using Nextera technology, further cleaned as above, and pooled at equimolar concentration. The final library was denatured with 0.2 N NaOH and diluted to 6 pM with a 20% PhiX control. Paired-end sequencing was performed on an Illumina MiSeq platform (Illumina, San Diego, CA), as per manufacturer’s guidelines, at Wellmicro Srl (Bologna, Italy).

### Bioinformatics and statistics

Raw sequences were processed using a pipeline combining PANDAseq^78^ and QIIME^79^. Length- and quality-filtered reads were clustered into Operational Taxonomic Units (OTUs) at 97% similarity using UCLUST^80^. All singleton OTUs and chimeras were discarded. Taxonomy assignment was conducted using the RDP classifier against the Greengenes database (May 2013 release). For species-level identification, OTUs of interest were subjected to BLAST analysis.

Publicly available sequences of the gut microbiota from age- and sex-matched healthy Italian subjects were downloaded and processed as above for comparative purposes. Specifically, we recovered sequences from De Filippis et al.^40^ (76 Italians; NCBI SRA SRP042234), Turroni et al.^41^ (1 Italian; NCBI SRA PRJNA340060), Schnorr et al.^42^ (8 Italians; MG-RAST mgp12183) and Biagi et al.^43^ (15 elderly Italians; MG-RAST mgp17761). Alpha diversity was calculated using the inverse Simpson index. Principal Coordinates Analysis (PCoA) was performed on Bray–Curtis distances between the genus-level profiles.

Statistical analysis was performed in R 3.3.2 (https://www.r-project.org/) using the packages ‘made4’^81^, ‘vegan’^82^ and ade4^83^. A permutation test with pseudo-F ratios (function ‘adonis’ in vegan) was used to assess the significance of separation in PCoA. The contribution of covariates to the ordination space was determined using the function ‘envfit’ of vegan. Wilcoxon rank sum test was used to assess significant differences in diversity and taxon relative abundance between groups while Kruskal–Wallis test was used for multiple comparisons. The impact of clinical variables on microbiota structure was also evaluated by Random Forests^84^, using the packages RandomForest and rfPermute function. Stratification into enterotypes was achieved following the enterotyping tutorial provided in the R environment by EMBL (http://enterotype.embl.de/enterotypes.html). Briefly, samples were clustered based on genus-level relative abundances using the Jensen-Shannon distance (JSD) and the Partitioning Around Medoids (PAM) clustering algorithm. The optimal number of clusters was chosen by maximizing the Calinski–Harabasz (CH) index. Cluster validation was performed using the silhouette coefficient. The results of clustering were visualized on a PCoA plot by the ade4 package. When appropriate, p values were adjusted for multiple comparisons using the Benjamini–Hochberg correction. A false discovery rate (FDR) < 0.05 was considered as statistically significant.

### Ethical declarations

The study was approved by the institutional ethical board (Comitato Etico Interaziendale Bologna-Imola) of the coordinating center (Montecatone Rehabilitation Institute), and subsequently by all participating centers (University of Bologna, ASST Gaetano Pini CTO, ICS Maugeri Bari, Ospedale Valduce, ICS Maugeri Pavia, Ospedale Santa Corona, Ospedale Cannizzaro, Azienda Ospedaliera di Perugia, Fondazione Teresa Camplani Domus Salutis Brescia, Ospedale Policlinico Bari), and conducted in accordance with the Declaration of Helsinki. All study participants completed a written consent form prior to their inclusion in the study.

## Supplementary Information

Supplementary Information.

## Data Availability

Sequence reads were deposited in the National Center for Biotechnology Information Sequence Read Archive (NCBI SRA; BioProject ID PRJNA724686).
