# Supplementary material for: Changes in gut microbiota in the acute phase after spinal cord injury correlate with severity of the lesion
Source: Sci Rep. 2021 Jun 17;11:12743. doi: 10.1038/s41598-021-92027-z (PMC8211659; doi:10.1038/s41598-021-92027-z)
Supplement: Supplementary file 1 — Supplementary Information. [file 41598_2021_92027_MOESM1_ESM.pdf]

Changes in gut microbiota in the acute phase after spinal cord injury correlate with severity of the lesion

Gabriele Bazzocchi, Silvia Turroni, Maria Chiara Bulzamini, Federica D'Amico, Angelica Bava, Mirco Castiglioni, Valentina Cagnetta, Ernesto Losavio, Maurizio Cazzaniga, Laura Terenghi, Luisa De Palma, Giuseppina Frasca, Beatrice Aiachini, Sonia Cremascoli, Antonino Massone, Claudia Oggerino, Maria Pia Onesta, Lucia Rapisarda, Maria Cristina Pagliacci, Sauro Biscotto, Michele Scarazzato, Tiziana Giovannini, Mimosa Balloni, Marco Candela, Patrizia Brigidi and Carlotte Kiekens

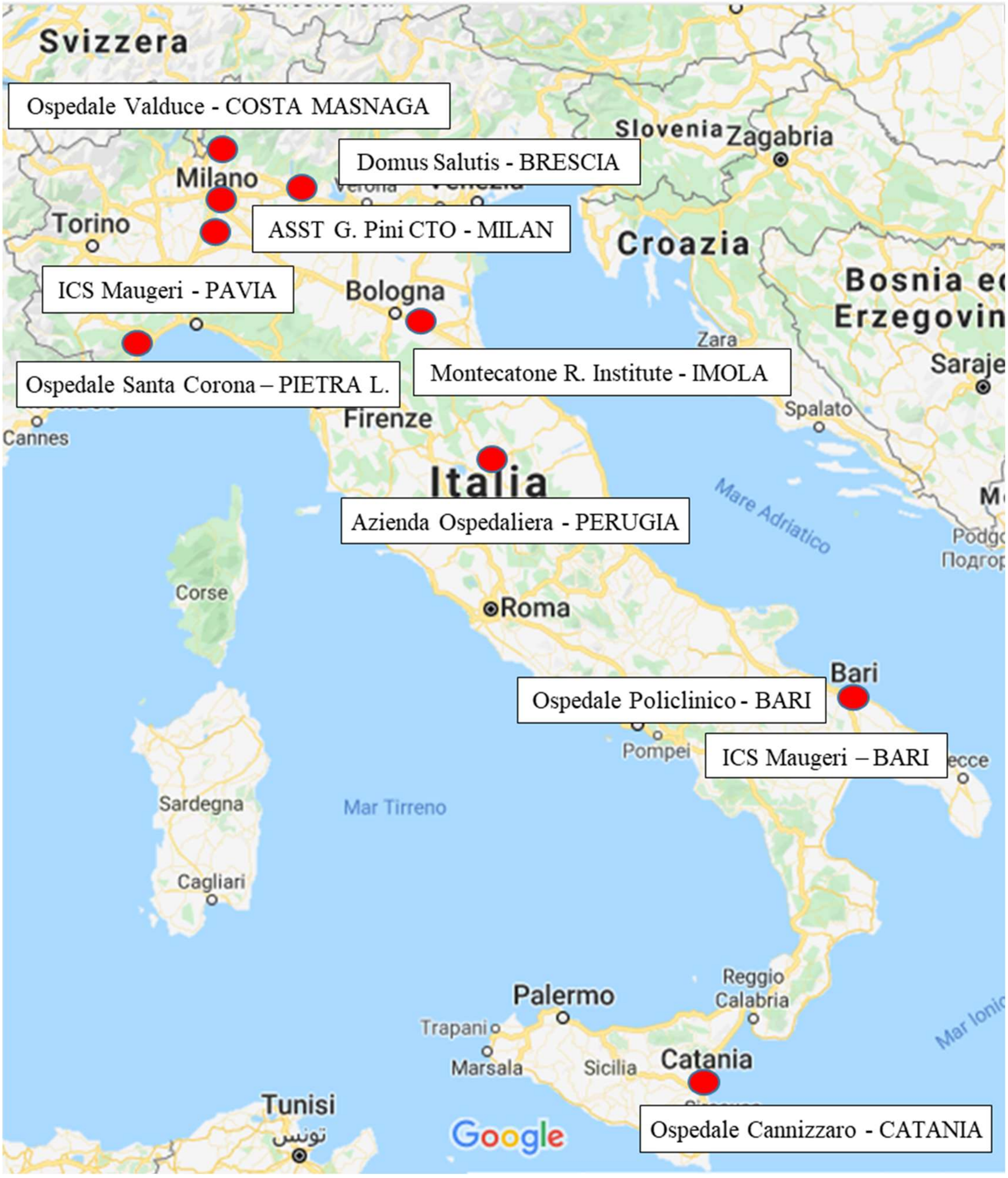

Figure S1. Geographic distribution of the 10 Spinal Units in Italy. The recruiting centers are equally located in Northern, Central and Southern Italy. The number of patients is also well distributed, with 31, 30 and 39 patients enrolled in the three Italian areas, respectively. The image was obtained from Google Maps and modified. See also Table 1.

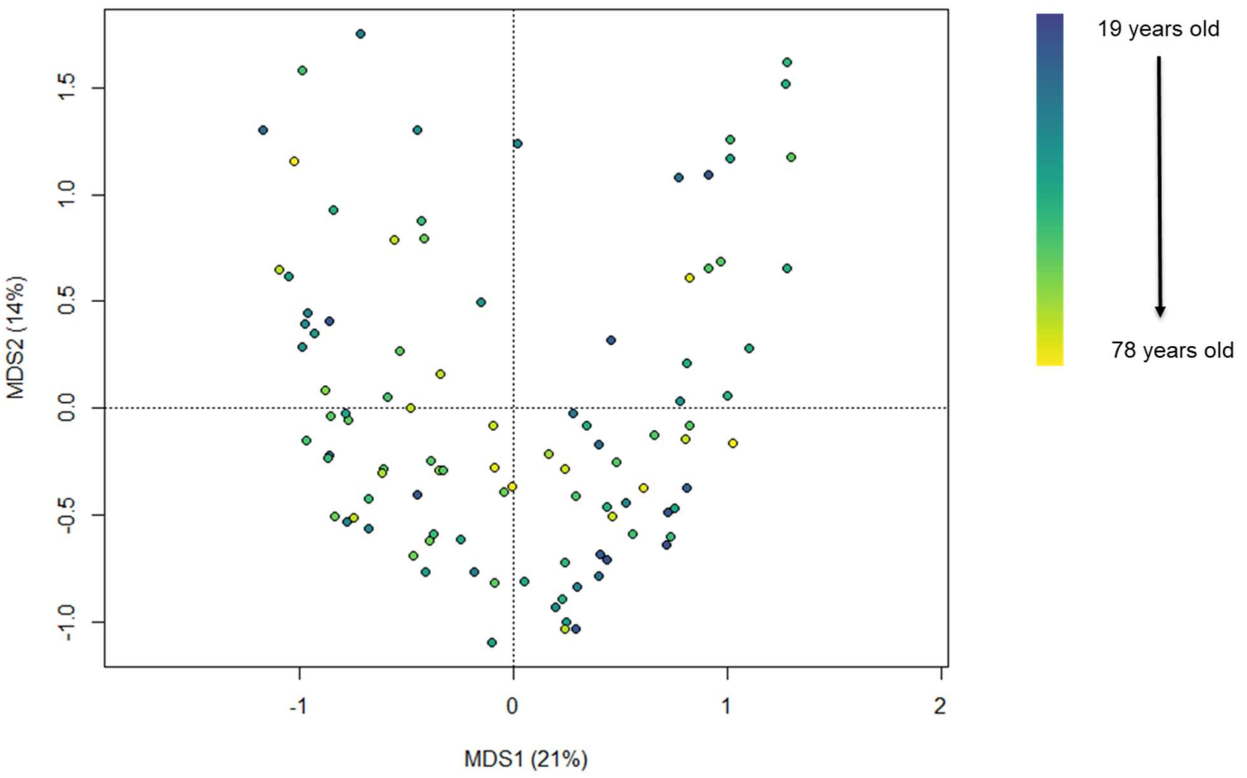

Figure S2. The gut microbiota dysbiosis in SCI patients is independent of age. Principal Coordinates Analysis based on Bray-Curtis distances between the genus-level microbial profiles of SCI patients. Dots are colored by subject age (see the color gradient on the right).

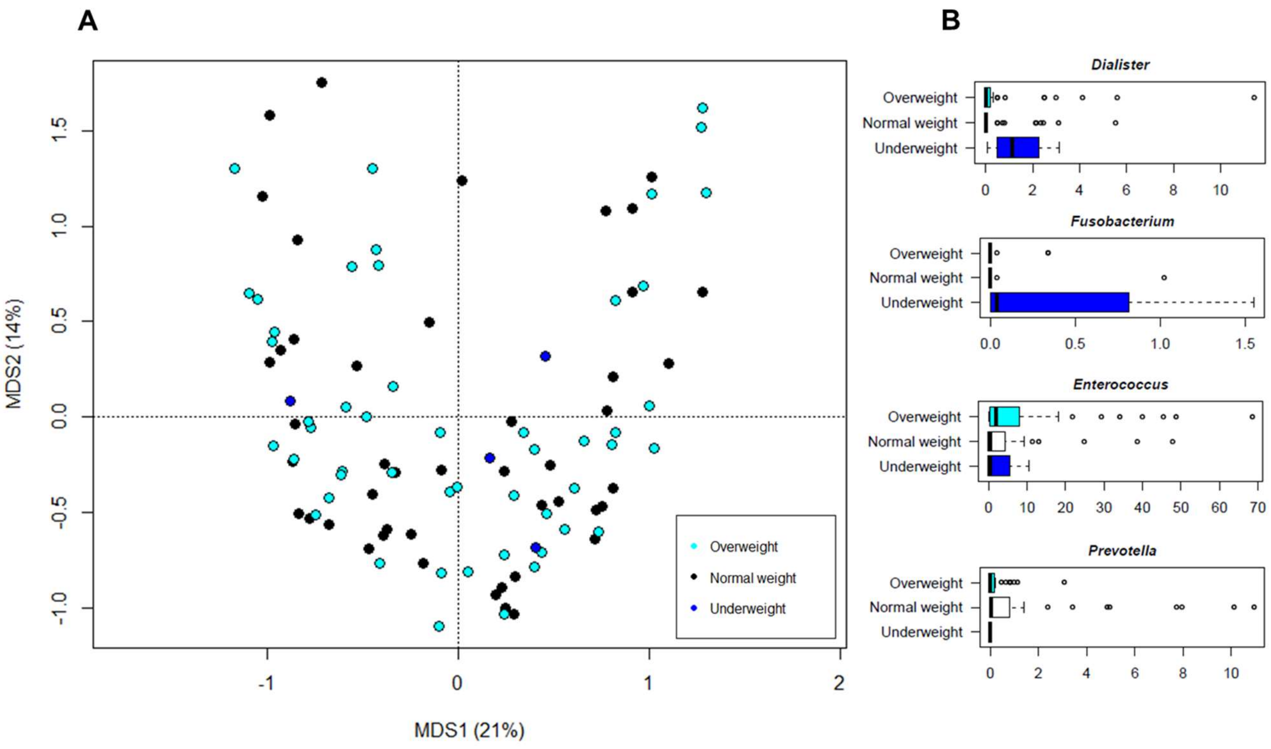

Figure S3. The gut microbiota dysbiosis in SCI patients is independent of BMI. A, Principal Coordinates Analysis based on Bray-Curtis distances between the genus-level microbial profiles of SCI patients according to BMI (under, normal or overweight). No significant segregation was found ($p>0.05$, permutation test with pseudo-F ratios). **B**, Box plots showing the distribution of the relative abundance values of genera differently represented among categories ($p\leq0.05$, Kruskal-Wallis test).

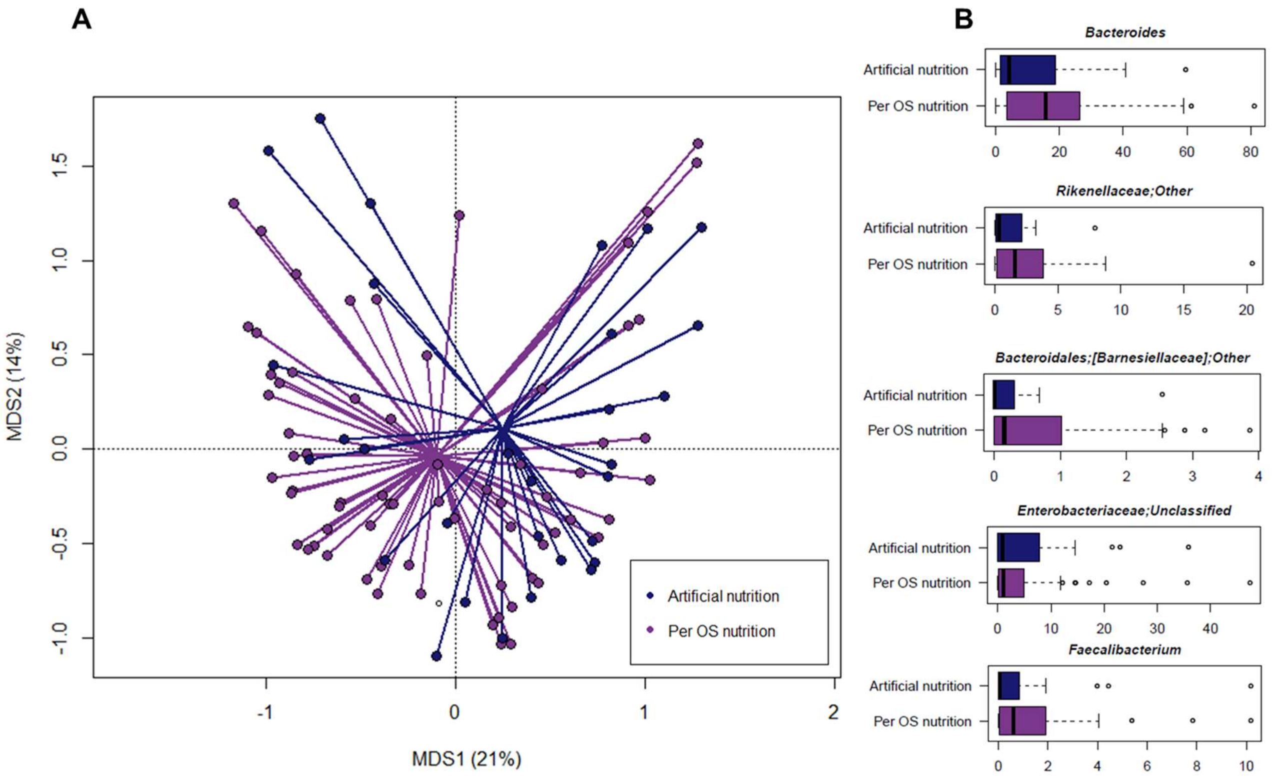

Figure S4. The gut microbiota dysbiosis in SCI patients is independent of the type of nutrition. A, Principal Coordinates Analysis based on Bray-Curtis distances between the genus-level microbial profiles of SCI patients according to nutrition type (*per os* vs. artificial, i.e. enteral/parenteral). No significant segregation was found ($p>0.05$, permutation test with pseudo-F ratios). **B**, Box plots showing the distribution of the relative abundance values of genera differently represented between categories ($p\leq0.05$, Wilcoxon rank sum test).

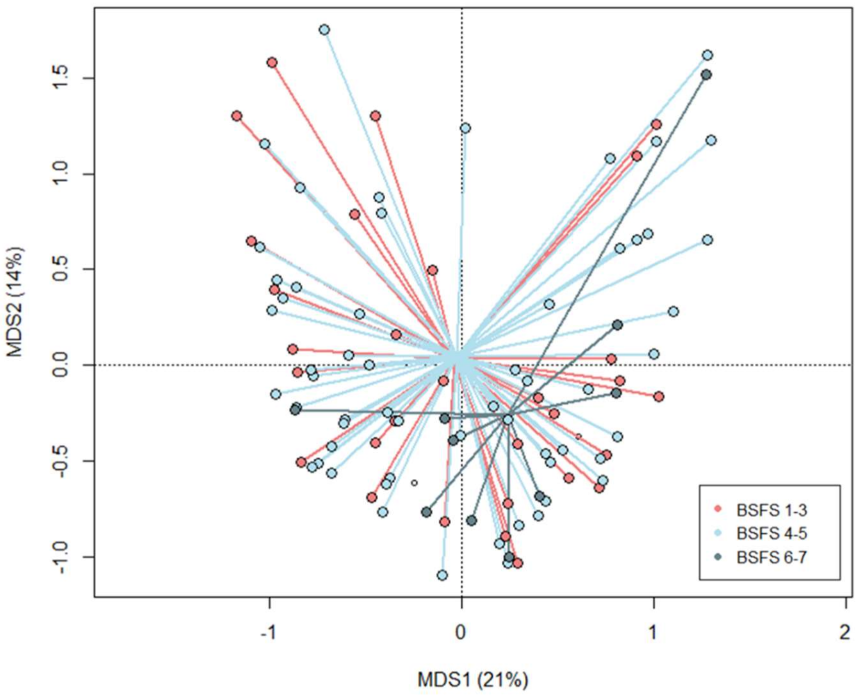

Figure S5. The gut microbiota dysbiosis in SCI patients is independent of Bristol Stool Form Scale. Principal Coordinates Analysis based on Bray-Curtis distances between the genus-level microbial profiles of SCI patients according to Bristol Stool Form Scale (BSFS 1 to 3 vs. 4-5 vs. 6-7). No significant segregation was found ($p>0.05$, permutation test with pseudo-F ratios).

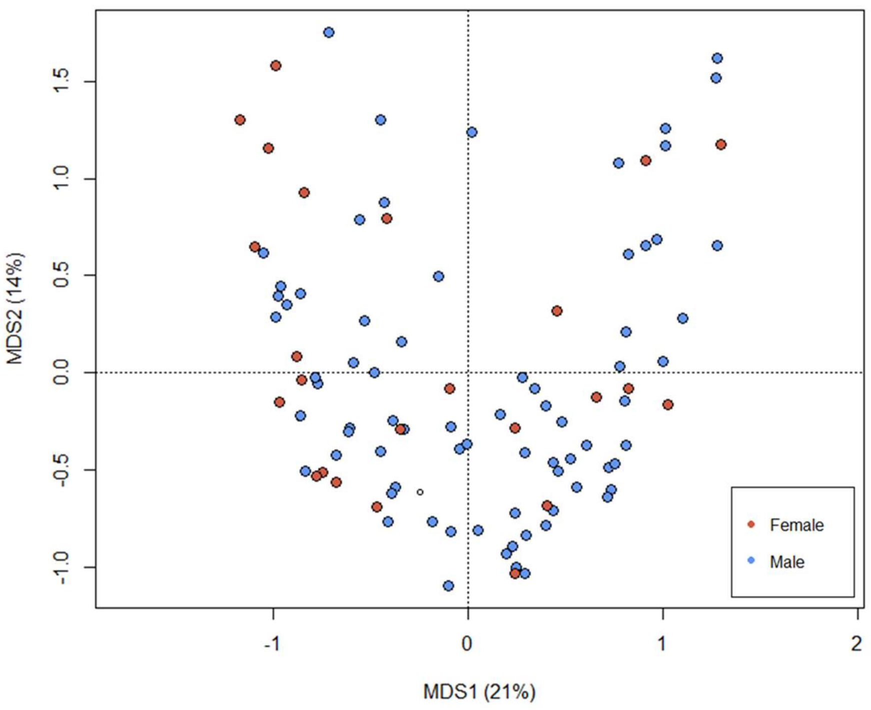

Figure S6. The gut microbiota dysbiosis in SCI patients is independent of gender. Principal Coordinates Analysis based on Bray-Curtis distances between the genus-level microbial profiles of SCI male vs. female patients. No significant segregation was found ($p>0.05$, permutation test with pseudo-F ratios).

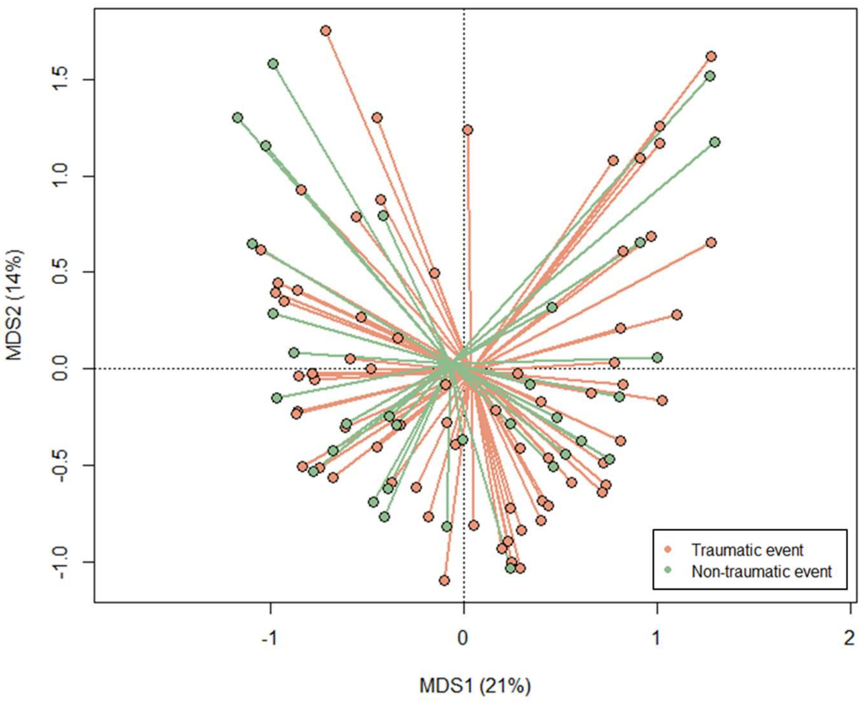

Figure S7. The gut microbiota dysbiosis in SCI patients is independent of the etiology of the lesion. Principal Coordinates Analysis based on Bray-Curtis distances between the genus-level microbial profiles of SCI patients undergone a traumatic vs. non-traumatic event. No significant segregation was found ($p>0.05$, permutation test with pseudo-F ratios).

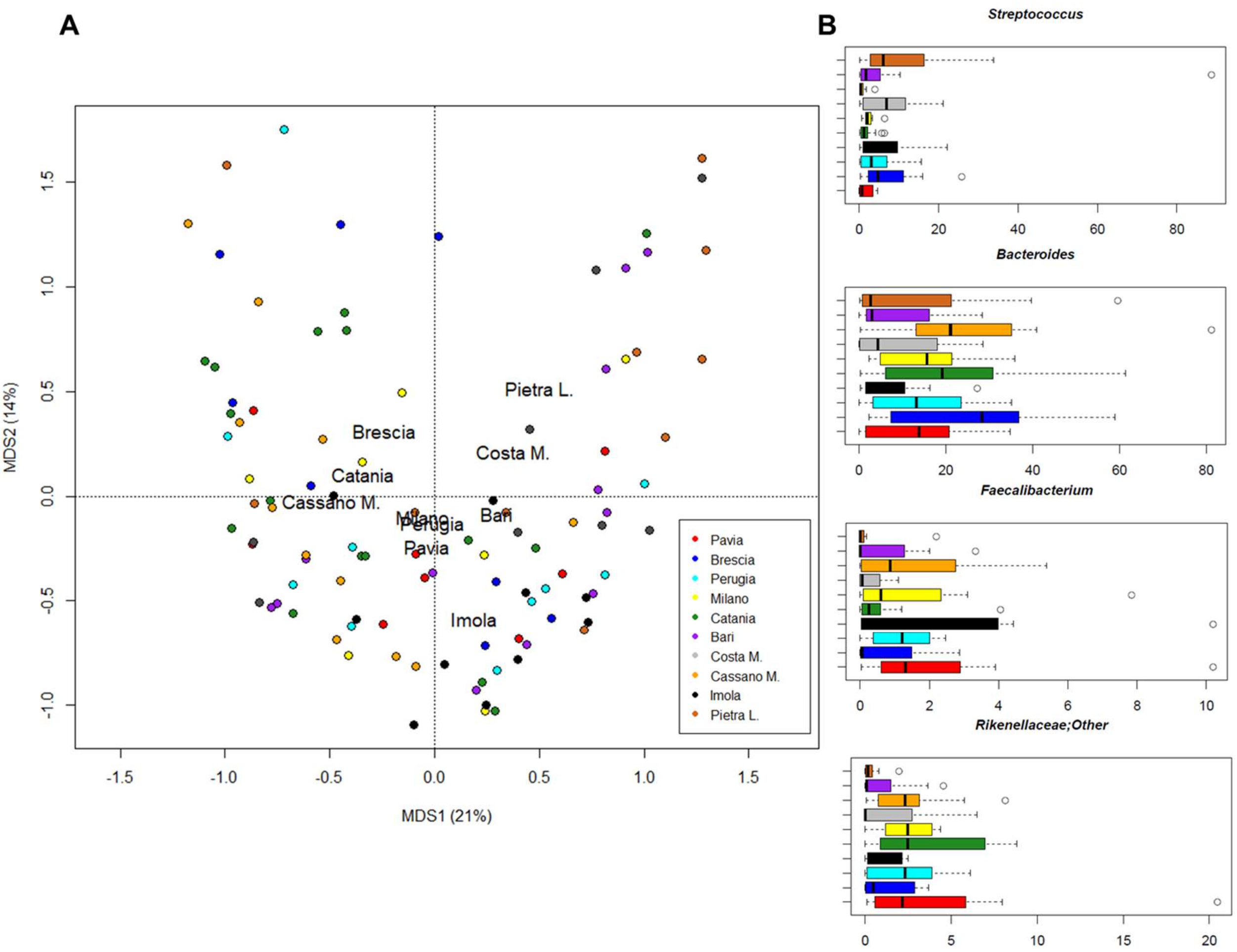

Figure S8. The recruitment center has only a slight impact on the gut microbiota structure. A, Principal Coordinates Analysis based on Bray-Curtis distances between the genus-level microbial profiles of SCI patients. A significant separation by spinal unit was found (p=0.003, permutation test with pseudo-F ratios). The centers are identified with the name of the respective city (Costa M., Costa Masnaga; Pietra L., Pietra Ligure) except for the spinal units of Bari (Bari, Ospedale Policlinico; Cassano M., ICS Maugeri). See also Figure S1. **B**, Box plots showing the distribution of the relative abundance values of rehabilitation unit-discriminating genera. $p \leq 0.05$, Kruskal-Wallis test.
